# A Guide for Medical Educators: How to Design and Implement In Situ Simulation in an Academic Emergency Department to Support Interprofessional Education

**DOI:** 10.7759/cureus.14965

**Published:** 2021-05-11

**Authors:** Derek L Monette, Daniel D Hegg, Angela Chyn, James A Gordon, James K Takayesu

**Affiliations:** 1 Emergency Medicine, Massachusetts General Hospital/Harvard Medical School, Boston, USA; 2 Emergency Medicine, Cambridge Health Alliance/Harvard Medical School, Cambridge, USA

**Keywords:** in situ simulation, interprofessional education, medical education, healthcare simulation, emergency medicine, healthcare teams

## Abstract

In situ simulation (ISS) put simulation training directly into the clinical practice environment. Although ISS creates opportunities to identify latent system threats, understand culture, and improve team dynamics, there are limited resources for medical educators to guide the development and implementation of ISS at academic (or community-based) emergency departments (EDs). We describe the implementation of ISS in a high-volume urban ED to help educators understand the requirements and limitations of successful program design. During an academic year, 66 individual learners participated in at least one of our 22 training sessions, a cohort that included 37 nurses, 17 physicians, eight physician assistants, and four allied health professionals. Feedback from these participants and case facilitators informed our iterative process of review and development of program guidelines and best practices. We share these key technical points and the themes we found to be essential to the successful implementation of an ISS program: consideration of session timing, participant buy-in, flexibility, and threats to professional identity. Overall, our report demonstrates the feasibility of implementing an ISS program in a high-volume urban ED and provides medical educators with a guide for creating an ISS program for interprofessional education.

## Introduction

The complexity of healthcare requires the coordination of interprofessional (IP) teams to deliver high-quality care. This is particularly true for emergency medicine (EM), where IP teams manage high-acuity patients using a variety of resources in a time-critical setting [1]. In situ simulation (ISS) takes place in the actual clinical space where patient care occurs, enabling team training using real-world equipment, physical spaces, and resources.

There is growing evidence that ISS may improve patient outcomes and team performance [2-4]. ISS can identify threats to patient safety and influence the relational aspects of patient care, helping teams identify shared goals and build mutual respect [5,6]. However, a minority (15%) of ISS initiatives involve emergency departments (EDs) [3]. Barriers to ED-based ISS include the unpredictable patient volume and acuity, limited clinical space, and potential risks to patient and provider safety. Furthermore, there are limited resources for EM educators to guide the practical development and implementation of a successful ISS program to support IP education and teams [7-10]. We designed our program to address this knowledge gap.

The educational goals of this program are for IP teams of EM physicians, nurses, advanced practice providers, and other health professions (e.g., respiratory therapists) to advance non-technical skills (e.g., closed-loop communication) and perform deliberate practice of low-frequency, high-risk patient encounters. The objective of this technical report is to provide educators with programmatic details and a guide for implementing a successful IP ISS program in the ED.

## Technical report

Our development phase (July 2017 to September 2018) focused on building consensus around learning goals and a safe approach to implementing ISS sessions. We describe the following steps and our ISS protocols in detail.

Identifying stakeholders

We identified key stakeholders in patient care and continuing education in our department by asking ourselves who will be impacted by this project and reviewing stakeholders from past educational innovations [11]. Our stakeholder list included: (1) clinical leadership (physicians, nursing, physician assistants [PAs], pharmacy); (2) education leadership (simulation instructors and designated clinical educators); (3) Quality and Safety Committee; and (4) our Operations Committee.

A core team with representation from each stakeholder group identified department learning needs, optimal physical space and timing for the simulations, and protocols for safe equipment use. This team consisted of two or more representatives from each stakeholder group and at least one representative from each clinical role to ensure an IP perspective. This team continued to refine the ISS educational content and structure during a series of pilot sessions and the implementation period (October 2018 to June 2019).

Defining goals

Stakeholders developed program goals through consensus after reviewing existing ISS literature and materials from laboratory-based simulation programs within the residency [11]. Representatives from each stakeholder group had an opportunity to propose long-term objectives for the program. After discussion, the group agreed to support the following goals: (1) developing a shared understanding of priorities in clinical management across IP teams; (2) improving communication skills to improve the efficiency and effectiveness of care; and (3) identifying latent systems threats to patient safety and opportunities for systems improvement.

Case development and instructors

Experienced simulation faculty with formal training in debriefing methods designed cases incorporating content from departmental clinical guidelines, quality assurance measures, and institutional case experience. Clinical topics included sepsis, hemorrhagic shock, tachyarrhythmias requiring cardioversion, and a variety of pediatric emergencies (e.g., respiratory distress). Pediatric emergency medicine (PEM) faculty designed and co-facilitated PEM sessions. Each session included a pre-brief (5 minutes), simulation (20 minutes), and structured debriefing (20 minutes).

Scheduling/use profile

Using historical ED census data, we identified times when the ED census is below capacity to hold ISS sessions (Monday 06:00 am to 09:00 am). For all sessions, an IP team of at least one EM resident physician and one nurse participated in a simulated case. The residents participated voluntarily, and they were not concurrently scheduled to work clinically in the ED at the same time. All other role groups participated during their scheduled clinical shifts, but they were free of clinical responsibilities for the duration of the case. The scheduled ED clinical team (e.g., resident and attending physicians) caring for patients in the ED could interrupt the simulation session at any point if the room was needed for patient care.

Access to ISS ED bay and equipment

A locking curtain was incorporated into the ED bay designated for ISS and mannequin storage. This room was a standard ED bay with patient monitors, a defibrillator, and equipment for airway management and intravenous (IV) access. Only designated “super-users” were permitted to unlock and remove this equipment. The super-users were also responsible for sequestering any used equipment from the patient care environment and returning the room to a patient-ready state at the end of the session. After session completion, the ED bay was restocked and cleaned in a standard fashion as if an actual patient had been cared for to ensure environmental safety (see below).

Code carts and defibrillators

An extra pediatric and adult code cart was designated for training only and stored outside the clinical area. Although for education use only, these carts were supported and restocked with actual medications and equipment according to hospital-based protocols after each session. This approach ensured training fidelity, protected the inventory of existing code carts, and guaranteed that each cart was fully functional at all times.

Airway equipment and procedure carts

ISS participants used the ED bay airway cart as needed for ISS cases. After each session, super-users would communicate with facilities management to ensure that the cart was checked and restocked per routine protocol prior to the bay returning to active clinical use.

Specialized durable equipment

Some additional specialized equipment (fiberoptic scope, C-MAC® laryngoscopes [Karl Storz, Tuttlingen, Germany], and EZ-IO® guns [Teleflex Medical Research, Triangle Park, NC, USA]) was acquired, designated, and labeled as “training only” for recurrent ISS use. This was done to protect the clinically deployed equipment from overuse and to ensure that critical equipment was never diverted from the clinical inventory. Only super-users had access to this equipment, locked and secured behind the partition with the mannequin. These were not removed from the bay and must be immediately secured at the end of the case.

Medications

Any medications from the Code Cart were always real unless there was a medication shortage that applied to all code carts across the institution (e.g., sodium bicarbonate) [12]. Proper use and disposal were coordinated with ED pharmacy. These real medications were drawn from the existing supply and disposed of per routine clinical protocol. All other medications were empty medication syringes and vials from the locked and secure bin. These empty medication syringes and vials were taped to each other and never reconstituted with any solution (e.g., water, saline) or air. Providers practiced connecting the syringe to the IV (without any injection), which created opportunities to perform closed-loop communication (e.g., “I’ve administered 4 milligrams of Zofran”) whether a medication was from the Code Cart or not. These empty syringes/vials could be used recurrently for convenience but remained locked and secured behind the storage partition after each session. Only super-users had access to these items and provided them to participants when requested. Super-users ensured these syringes/vials were always empty and returned to a locked and secure bin.

Fluids

Intravenous fluid (IVF) bags were drawn from the actual stock supply. The only exemption was if there was a shortage of any type of IVFs. For example, while implementing our program, there was a shortage of IV normal saline [13]. During this period, a completely empty IVF bag was used to “represent” IVF administration. Only super-users had access to these labeled empty IVF bags and could provide them to participants when requested. Super-users also disposed of any partially filled IVF bags at the end of a session. Altering these bags or refilling them (e.g., with water) to “simulate” actual conditions was prohibited in order to minimize the risk of administering these IVF bags to patients after the simulation.

Blood products

Real blood bank products are never used in the simulation. Simulated blood could be used only if approved in advance by the core team, working in collaboration with the central blood bank. In such cases, the blood bank prepared and delivered the simulation bags according to previously established protocols for such drills. A simulation-only Belmont infuser was available and labeled as “training-only” and secured behind the partition.

Achieving buy-in and recruitment

Representatives from each stakeholder group served as advocates for the initiative and approved staff participation. Nursing leadership communicated with the ED resource nurse the evening before each session to assess department volume and acuity. All provider role groups including physicians, nurses, residents, PAs participated on a voluntary basis, as facilitated by respective leadership. A general topic invitation was used to announce the session (e.g., “please join us to review the management of hypotension”), withholding the specific diagnosis (e.g., “urosepsis”). We used recent challenging cases in the department or topics that staff and/or trainees requested to increase buy-in and interest in participation. Residents were also awarded Individualized Interactive Instruction (III) credit for their participation [14].

Outcomes

**Figure 1:**
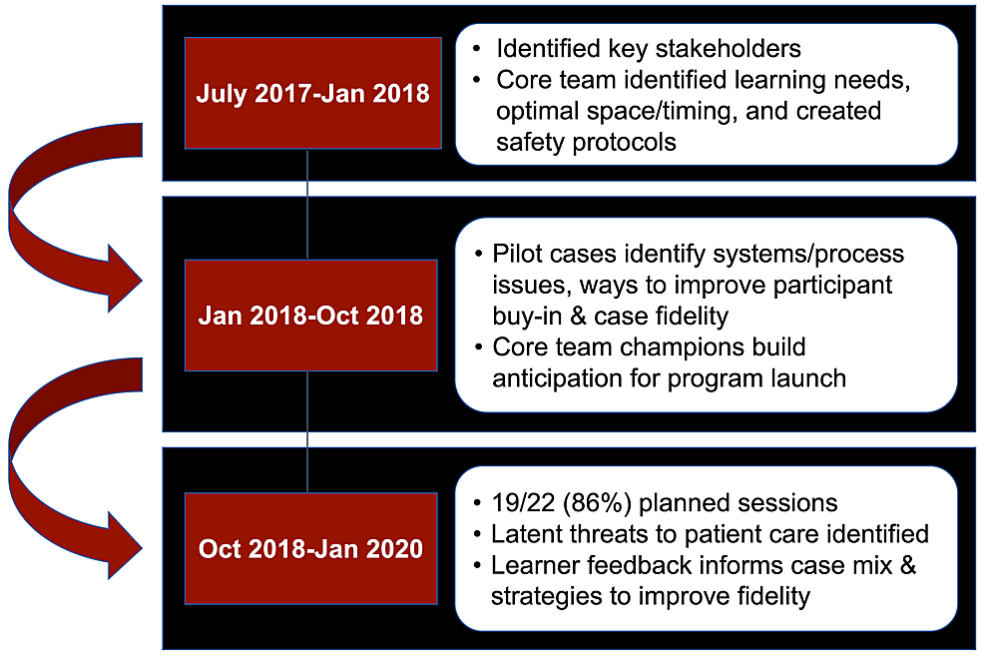
A graphic representation of the in situ simulation program’s development and implementation timeline.

Figure 1 reviews our initiative’s timeline from planning and design to implementation. We evaluated the success of this program by (1) calculating the percent of scheduled simulation sessions that were completed without cancellation or delay and (2) recording the distribution of role groups reflected in our learner cohort. These metrics were collected because a high completion rate reflects the feasibility of the program and a significant IP learner cohort was a programmatic educational goal. Between October 2018 and January 2020, we completed 19/22 (86%) of scheduled IP ISS sessions. A total of 66 individual learners participated in at least one session, a cohort that included 37 nurses (57%), 17 physicians (26%), eight PAs (12%), and four allied health professionals (6%). We did not set a specific goal for the number of participants from each role group; however, the proportion of each role group in our learner cohort approximates the makeup of our ED treatment teams. Finally, we canceled three (14%) sessions either because the ED census was above capacity or because we were unable to schedule representation from more than one role group. There were no patient safety issues or critical events reported as being related to the simulation.

## Discussion

We provide medical educators with a detailed guide for creating an ISS program to support IP education in the ED. Table 1 summarizes key considerations and practical points we have learned in developing this program. These reflect topics that each of the authors considers central to ISS design, implementation, or both.

**Table 1:** Key considerations for designing and implementing ISS in an ED. ED, emergency department; EMR, electronic medical record; ISS, in situ simulation

Key Consideration	Description
Physical layout	We re-configured a designated ED bay to store a simulation mannequin for easy deployment and storage. Others may consider storing a mannequin or task-trainer in a secure nearby location or the use of standardized patients.
Timing	We recommend using EMR to incorporate trends in patient volume and department staffing to determine the optimal timing of ISS sessions.
Content	Consider ways to align department goals with learner motivations. For example, we created a sepsis case that provides residents with an opportunity to practice airway management while also reviewing a new department quality initiative around time to antibiotics. Debriefings may be structured to review these fundamentals while also exploring systems of practice.
Participant buy-in and involvement	High-risk, low-frequency presentations or procedures seem to promote participant buy-in. Facilitators can increase the likelihood that participants identify the simulation as an important educational experience if they structure “future application” (of new knowledge) into the debriefing.
Resources and supplies	We treated each simulation as a patient encounter; supplies and equipment used in the simulations were taken directly from the ED supply carts or from “extra” inventory (e.g., same model) designated specifically for this purpose. This approach maximizes fidelity of the simulation and increases the likelihood of identifying latent safety threats (e.g., variable familiarity with specialized equipment). Real medication/infusion is always encouraged; when not feasible, critical safety rules apply in the ISS setting.
Flexibility and scalability	Both instructors and learners must be flexible, acknowledging that the surrounding patient care environment may influence the experience of the simulation. Consider creating cases that can be scaled in terms of complexity depending on the team’s performance and unexpected events (e.g., an unstocked supply cart).
Threats to professional identity	Invest in the pre-brief and establish a safe learning environment. Learners may fear exposing a knowledge gap in the very environment in which they provide actual patient care. Facilitators should be familiar with the concept of psychological safety and seek to establish an environment in which learners feel comfortable taking risks (e.g., perform introductions and establish confidentiality agreement across participants) [15,16].
Establish shared educational goals	Facilitators can level the playing field for interprofessional teams by displaying leader inclusiveness in the pre-brief [17]. Acknowledge that both facilitators and participants in the simulation are there to learn with and from each other. Facilitators can identify shared educational goals (e.g., “let’s review how together we can improve the care we provide patients with sepsis”) and frame the simulation as a collaborative learning experience for each participant.

While implementation strategies need to be locally tailored to the clinical environment and available resources, this program is most likely to be successful if educators focus on the following three points: (1) incorporating routine protocols that already exist in one's ED (e.g., protocol for restocking equipment after patient care) to ensure safe resource management; (2) creating participant buy-in with high-risk and/or low-frequency cases; and (3) developing strategies to mitigate threats to professional identity that may be inherent to ISS.

Other programs may increase their chances for success in implementing ISS by collaborating with department leaders in quality and safety. Our cases related to sepsis and pediatric emergencies support quality improvement by providing teams with opportunities to identify latent systems threats to patient safety. For example, simulations related to sepsis led to discussions between clinicians and nurses about the barriers to timely administration of broad-spectrum antibiotics. This natural link to quality and safety is underscored by previous studies of ISS programs in pediatric EDs and hospital systems [5,18].

Finally, the successful design of an ISS program is dependent on a strong focus during the development phase on a plan to ensure provider and subsequent ED patient safety. We recommend treating the simulation as an actual patient encounter and building off current local ED safety protocols.

## Conclusions

In summary, ISS is a valuable educational intervention that can be deployed in a busy academic ED. It requires stakeholder commitment to developing learning goals and safe implementation. In addition to harnessing pre-established protocols, programs should designate specific individuals responsible for ensuring that the ED bay remains a safe environment as it transitions from a training space back into a routine clinical area. Our report provides educators with guidelines for navigating the logistical and operational challenges of pursuing ISS in a busy ED. Future work may focus on developing best practices for pre-briefing and debriefing IP teams in ISS, improving learner buy-in, and identifying strategies to manage threats to professional identity inherent to IP ISS.

## References

[1] Olde Bekkink M, Farrell SE, Takayesu JK (2018). Interprofessional communication in the emergency department: residents' perceptions and implications for medical education. Int J Med Educ.

[2] Andreatta P, Saxton E, Thompson M, Annich G (2011). Simulation-based mock codes significantly correlate with improved pediatric patient cardiopulmonary arrest survival rates. Pediatr Crit Care Med.

[3] Miller D, Crandall C, Washington C 3rd, McLaughlin S (2012). Improving teamwork and communication in trauma care through in situ simulations. Acad Emerg Med.

[4] Rosen MA, Hunt EA, Pronovost PJ, Federowicz MA, Weaver SJ (2012). In situ simulation in continuing education for the health care professions: a systematic review. J Contin Educ Health Prof.

[5] Patterson MD, Geis GL, Falcone RA, LeMaster T, Wears RL (2013). In situ simulation: detection of safety threats and teamwork training in a high risk emergency department. BMJ Qual Saf.

[6] Brazil V, Purdy E, Alexander C, Matulich J (2019). Improving the relational aspects of trauma care through translational simulation. Adv Simul (Lond).

[7] Kurup V, Matei V, Ray J (2017). Role of in-situ simulation for training in healthcare: opportunities and challenges. Curr Opin Anaesthesiol.

[8] Spurr J, Gatward J, Joshi N, Carley SD (2016). Top 10 (+1) tips to get started with in situ simulation in emergency and critical care departments. Emerg Med J.

[9] Petrosoniak A, Auerbach M, Wong AH, Hicks CM (2017). In situ simulation in emergency medicine: moving beyond the simulation lab. Emerg Med Australas.

[10] Yager PH, Lok J, Klig JE (2011). Advances in simulation for pediatric critical care and emergency medicine. Curr Opin Pediatr.

[11] Binstadt ES, Walls RM, White BA (2007). A comprehensive medical simulation education curriculum for emergency medicine residents. Ann Emerg Med.

[12] Thompson CA (2017). Sodium bicarbonate shortage found to affect hospitals' daily operations. Am J Health Syst Pharm.

[13] Sacks CA, Kesselheim AS, Fralick M (2018). The shortage of normal saline in the wake of hurricane Maria. JAMA Intern Med.

[14] (2017). ACGME program requirements for graduate medical education in emergency medicine. http://www.acgme.org/Portals/0/PDFs/FAQ/110_emergency_medicine_FAQs_2017-07-01.pdf.

[15] Edmondson A (1999). Psychological safety and learning behavior in work teams. Adm Sci Q.

[16] Kolbe M, Eppich W, Rudolph J (2020). Managing psychological safety in debriefings: a dynamic balancing act. BMJ STEL.

[17] Nembhard IM and Edmondson AC (2006). Making it safe: the effects of leader inclusiveness and professional status on psychological safety and improvement efforts in health care teams. J Organiz Behav.

[18] Barbeito A, Bonifacio A, Holtschneider M, Segall N, Schroeder R, Mark J (2015). In situ simulated cardiac arrest exercises to detect system vulnerabilities. Simul Healthc.

